# Mapping frameworks and approaches to measuring the quality of transition support services for young people with intellectual and developmental disabilities

**DOI:** 10.3389/fresc.2023.1043564

**Published:** 2023-02-21

**Authors:** Julie Beadle-Brown, Jan Šiška, Šárka Káňová

**Affiliations:** ^1^ Tizard Centre, University of Kent, University of West Bohemia in Pilsen, Canterbury, United Kingdom; ^2^ Faculty of Education, University of West Bohemia in Pilsen, Pilsen and Charles University, Prague, Czech; ^3^ Faculty of Education, University of West Bohemia in Pilsen, Pilsen, Czech

**Keywords:** intellectual and developmental disabilities, transition, quality of support services, quality of life, education, measurement

## Abstract

Transition to adulthood for young people with intellectual disabilities and developmental disabilities (IDD) has been given significant attention in research, policy development and practice. The aim of this paper was to explore how a recently developed theoretical outcomes-based framework for measuring the quality of services for people with disabilities could potentially be useful in conceptualizing and supporting successful transition to adulthood. The theoretical discussion draws on both the scoping review and template analysis that was used to develop the Service Quality Framework and on a separate study synthesizing expert completed country templates and literature review which included models of and research on successful transition to adulthood. Synthesis identified that using a quality of life outcomes focused framework of Service Quality could be mapped onto and extend current thinking on what is seen as successful transition to adulthood by putting the focus on successful transition as people with IDD moving towards having similar opportunities and quality of life as other adults without disabilities living in the same community/society. Implications of a more wide-ranging definition and holistic view for both practice and future research are discussed.

## 1. Introduction

Transition to adulthood for young people with intellectual and developmental disabilities (IDD) has been emphasized as an area of considerable significance. The difficulties this group experiences when moving from school to employment, to higher education and active participation in the community after leaving school, has been highlighted as a major contributor to isolation and exclusion (1). Despite the United Nations Convention for the Rights of Persons with Disabilities (2), young people with IDD and complex needs often transition from special schools to other congregated settings such as to care homes, because of the lack of appropriate alternatives (3, 4).

One key issue is that there is as yet no agreed conceptualization of what constitutes “successful transition” more generally or specifically for young people with IDD. Some of the literature has focused rather narrowly on transition from child to adult health, mental health and/or social care services (5, 6) or on transition predictors in-school activities that positively correlate with postschool success in post-secondary education, employment, and independent living (7). Some conceptualizations of transition have had a broader focus. Wehman (8) conceived of transition as a *period of significant life changes* that naturally occur after leaving school including change in the concept of oneself; body changes; sexuality and partnership; financial and work needs; the need for independence in travel and mobility, etc. Much of the literature focuses on transition as moving into adulthood and “successful transition” equates to achieving indicators of adulthood, such as employment, financial independence, post-secondary education, and engagement in close relationships such as marriage, or parenthood (9). However, some of these role transitions are becoming less reliable indicators of adulthood, as altering economic and social conditions continue to change the traditional path to adulthood for all youth, for example, due to difficulties with finding employment (10). This potentially impacts on whether young people can be financially independent and live independently, meet a wider range of people with whom to form relationships, etc. Factors that hinder and facilitate the participation of persons with IDD in employment are often found in the environment, with personal factors also influencing participation. The presence of negative attitudes and lack of support services were major limiting factors within the environment (11, 12). In addition, definitions of “success in employment” shared by parents of young persons with intellectual disabilities appear to go beyond the constrained criterion of full-time competitive employment. Parents value a range of occupational outcomes that consider their son or daughter's skills and interests, just one of which was competitive employment.

A recent review of policy, guidance and research focusing on four countries related to successful transition of young persons with IDD identified that, although successful transition is conceptualised in different ways in different countries (13), there appear to be five core outcomes or indicators of transition success:

• having a job (employment) and/or financial independence (the U.S., United Kingdom, the Czech Republic, and Australia),
• independent living/moving out of the family home (the U.S., Australia, and United Kingdom),
• further education (the U.S.),
• growing your social networks, relationships and being part of your community; (United Kingdom),
• physical and mental health/well-being (United Kingdom).

Such indicators of “successful” transition have been regarded by some as normative or even harmful, particularly for young disabled people, who often face additional and significant barriers to achieving these indicators (14, 15). For many people with disabilities, support is required through both, the process of transition typically to employment or independent living and into adulthood itself. The higher people's support needs are, the more help people are likely to need. This means that support services of one type or another are likely to be involved in the transition process. Unfortunately, research tells us that quite often services are not structured holistically—across the life span, seeing people across the threshold from child to adult environment (16). Reasons for it could be found for example in rigid fragmentation of support services into specific administration entities such as health, education, social security, and social services. This might result in a gap or risk of people falling through the gaps (17, 18). On the contrary, programs for helping young people with disabilities to develop the skills needed for adulthood only exist in a few countries and the focus of these is primarily on further education. Often, there is a primary focus on transition planning, which is used on a voluntary basis in most countries, although is embedded in federal disability legislation in the U.S.—Individuals with Disabilities Education Act, 2004.

There is also relatively little data on how successful transition programs and support are—both in terms of short term and long-term outcomes and on what might predict successful transition. Although there is some research on models of successful transition to adulthood for young people without disabilities (19) and some predictors of successful transition to adulthood of persons with disabilities have been identified (7, 20), much less is known about the factors that determine successful transition for young people with intellectual and developmental disabilities and how to measure quality of transition support provided with participation of those who use such support. One study from Scotland evaluated a personal budget scheme used in Scotland. The results suggest that giving people a transition related personal budget can be useful, however, having access to funding is only any use if you know what you want to buy and where (15). In other countries there are specific transition support services (sometime called transition programs) to prepare young people for adulthood, but these are frequently segregated rather than integrated or inclusive. In addition, Lindsay et al. (21) reported positive impacts from a range of interventions for young persons with IDD, but there was a gap in research focusing on the types of support that work best, and how young disabled people viewed these.

So, with limited data on the impact of transition programs, no agreed definition of what success looks like and a lack of systematic strategies for assessing impact of transition support, this makes it very difficult to both develop new services and evaluate existing services and support in terms of how well they promote successful transition to adulthood. In this paper, we draw on two separate research studies to discuss a potential theoretical model for thinking about successful transition for young persons with IDD and how this might be implemented and measured. The first study (22) focused on developing a framework that could be potentially used for measuring the quality of disability services across Europe (hereafter referred to the Service Quality Framework). The second study (13) collated research, policy and information on practice and support related to transition of young people with intellectual disabilities in four countries.

## 2. Methods

### 2.1. The development of the service quality framework

The development of a theoretical outcomes-focused framework for measuring service quality was commissioned by the European Association of Service Providers for People with Disabilities (EASPD) as a response to the new European Strategy on the Rights of Persons with Disabilities 2021–2030, which included the aim of developing a European Framework for Social Services of Excellence for Persons with Disabilities. The remit of the research was to develop a framework for measuring the quality of social services for people with disabilities and a set of quality indicators which were (a) in line with the UN CRPD and (b) focused primarily on quality-of-life outcomes. This process was seen as the first phase in an ongoing program of work that would ultimately empirically test the feasibility and reliability of the Service Quality Framework. Although the final version of the commissioned Framework (22) included domains that went beyond outcomes to include indicators of processes and structures (23), for the purposes of this paper we will focus primarily on the sections of the framework that focused on quality of life outcomes.

#### 2.1.1. Introduction to quality of life outcomes

The concept of quality of life has a long and varied history, with varying definitions and conceptualizations used over time. Key developments in the conceptualization of QOL and service-related outcomes that led up to the production of the international consensus on Quality of Life led by Schalock et al. (24) are summarized in Schalock and Verdugo (25) and discussed further in Šiška and Beadle-Brown (22). Although recognizing that there are other frameworks of quality of life such as the ICF framework, Šiška and Beadle-Brown (22, 26) note that the ICF framework is more commonly used with reference to health-related quality of life and is also focused at a much wider systems or societal level more generally. Whilst it is important to acknowledge the interactions between wider societal aspects and individual quality of life, it was felt that, in the context of monitoring the quality of social care services, it was important to have a framework which makes it clear how services can positively influence people's outcomes and reduce the likelihood that services will attribute poorer quality of life outcomes to societal or impairment related factors. Thus, this research used the eight quality of life domains set out in the international consensus of 2002 (24)—physical well-being, emotional well-being, material well-being, social relationships, social inclusion, personal development, self-determination and rights—and drew on the conceptualization most recently summarized in Schalock and Verdugo (25).

#### 2.1.2. The scope and methods of the service quality framework development work

The scope of the commissioned framework included the following:

• any service providing in-home support for living of any type to children or adults with disabilities living in their own home,
• any service providing short term care and support/respite/short breaks (in home or out of home)
• any service providing residential care for people with disabilities
• any service providing day activities, occupation, training for work or independent living, etc.

Services which were primarily providing support in health, education or in employment were not included in the research.

Two primary methods—a scoping review of the published literature (as described in the JBI Manual for Evidence Synthesis (27) and a template syntheses—were used to identify international literature, policy and frameworks related to measuring the outcomes of services.

#### 2.1.3. Scoping review

The scoping review focused on identifying and clarifying how service quality had been defined and measured in the published peer-reviewed and grey international literature.

##### 2.1.3.1. Inclusion criteria

Population—literature (including grey literature) related to people with disabilities (could include all disabilities and mental health problems).

Concept—Service quality—definitions, conceptualization and measurement. Had to include some reference to outcomes for people using services.

Context—literature (including grey literature) which explored quality of services for people with disabilities:

• in any country
• In the following types of services:
  ○ any service providing in-home support for living of any type to children or adults with disabilities living in their own home,
  ○ any service providing short term care and support/respite/short breaks (in home or out of home)
  ○ any service providing residential care for people with disabilities
  ○ any service providing day activities, occupation, training for work or independent living, etc.

Services which were primarily providing support in health, education or in employment were not included in the research.

Language—published in English.

Years—no limitation although if more than one version of a framework or model weas identified the most rest one was included.

Types of evidence—This was left open within the defined concept and context of the review to allow the findings to be as comprehensive as possible.

##### 2.1.3.2. Search strategy

Evidence was identified through three methods:1. Academic Publication Database search using EBSCO Host, Scopus and Web-of-Science

a. using the following string of search terms: Service quality AND Disab* AND Concept* OR Defin* OR Measur*.
b. Citation searches for “Donabedian”

1. a general Google search using the same search terms to identify sources not published in academic journals such as websites, policy or guidance etc.
2. the authors' existing knowledge, academic networks and the content of a recent book on Quality in Social Services (28).

##### 2.1.3.3. Quality assessment

Quality was not assessed as the review was identifying how service quality was defined and evaluated and we were interested in any frameworks or tools that were being used. As such sources were not exclude on the basis of quality. In reality, only a very small number of the models and frameworks identified had been evaluated empirically.

##### 2.1.3.4. Data extraction

Out of a total of 126 publications identified for the initial review, 40 publications (covering 14 countries) were identified for inclusion in the data extraction process for the scoping review. An additional, 34 publications were used to complete the country templates for the UK, Australia, and USA (see below). Data extraction focused on identifying the frameworks used to conceptualize and measure service quality, indicators of service quality and any domains used to organize these indicators, with a particular focus on outcomes. Please note that in this context the word “indicator” is used to mean something that indicates the state or level of outcomes. These are usually characteristics, artefacts or events that can be observed or that individuals might report in terms of their experiences. It is not used to imply statistical predictability.

#### 2.1.4. Template synthesis

For the template synthesis, a specifically designed template was used to gather and organize information from a range of 14 countries identified to represent different types of social service systems and contexts.. In the case of European countries, this template was initially sent to National Disability Experts for input who were part of European Disability Expertise network (EDE). Where no response was gained from the national experts, other contacts were approached, e.g., through European level umbrella organizations for service providers, social service directors and disabled people's organizations or family-based networks.

The country template was available in two formats—a detailed structured form guiding people with a list of questions to answer and a more open, descriptive format, if people felt there was limited information in the structured form, or they did not have sufficient time to complete the detailed version. Information about the project and instructions for completing the template were provided.

The template was designed to collate information on both formal (i.e., embedded in legislation) methods of defining and measuring quality and more informal measures, such as voluntary frameworks used by service providers, or disabled people's organisations.

Some of the country templates were completed by the research team using the information identified in the literature review specific to those countries and publicly available information (such as the DOTCOM EU disability database) and then checked with local experts where possible.

Relatively complete templates from the national experts were gained for eight countries: Germany, UK, Ireland, Romania, USA, Czech Republic, Finland, and Australia. In addition, some less detailed information was available from country experts and in written sources instead of the country templates for Sweden. Norway, Netherlands, Slovenia, and Spain.

The information gathered and organized in the templates was then reviewed and analyzed by the researchers with a focus on how service quality was conceptualized or defined, whether outcomes featured in these conceptualizations and if so, which quality of life domains were featured (even if quality of life was not specifically mentioned). The relationships with the UNCRPD were also explored. In addition, analysis focused on how service quality and outcomes are measured and whether people with disabilities and other stakeholders have been involved in the development of the frameworks and tools. Finally, innovative frameworks and tools that were in line with the objectives of this research were identified and synthesized into a separate datafile to draw out the dimensions of quality and outcomes included and how quality was measured.

#### 2.1.5. Evidence synthesis

The information gathered from both the scoping review and template synthesis was summarized and used to identify frameworks and tools which were used to conceptualize or measure service quality, and which included at least some element that focused on outcomes.

Table 1 Summarizes the literature identified, used and the countries covered by the literature.

**Table 1:** Summary of the literature identified by the scoping review and template analysis in the development of the service quality framework.

	Number of papers
Number of peer-reviewed academic papers identified as potentially relevant on title and abstract scan	31
Number of publications identified from other sources (including grey literature)	96
**Total identified for possible inclusion**	**126**
Number excluded completely on reading full text	35
Number identified as relevant to introduction/background	11
Number only relevant in terms of informing methodology for framework development (i.e., they were not related to social care settings or people with disabilities but looked at methodologies for developing frameworks). Excluded for the purpose of this paper	6
Number used for detailed country templates (UK, Australia and US and not used in the more general review)	14 (UK) 17 (Australia) 5 (USA) 34 Total
**Final number of papers, reports and other documents included in the review of literature on quality frameworks and indicators and data extracted**	**40**
Countries from which literature on Frameworks and Indicators was included	USA Australia UK Ireland Netherlands Sweden New Zealand Lithuania Europe (generally) Serbia Canada Czechia Spain Romania Greece

The 20 Frameworks identified from this strategy varied in terms of country of origin (USA, Australia, UK, Netherlands, Ireland, Czech Republic, and New Zealand; two were cross European measures). These 20 frameworks were then mapped onto the quality-of-life domains identified by the Schalock et al. (24) International consensus on quality of life (QoL). Only two of the identified frameworks used the domains directly. All the remaining frameworks included at least some elements that could be mapped onto at least one of the QoL domains, with some of the identified frameworks mapping to all of the QOL domains, either at the overarching domain level or at the level of individual indicators or standards. For example, on the Home and Community-based Services Outcomes (USA) National Quality Framework, the domain of social connectedness included items that mapped to both the Interpersonal Relationships and Social Inclusion domains of the QOL Framework. On the National standards for Residential services for children and adults with disabilities (Ireland), Standard 3.1 states that “Each person is protected from abuse and neglect and their safety and welfare is promoted”—this individual standard mapped onto the wider QOL domain of physical well-being. At the end of the mapping process, the 2002 QoL conceptualization was found to still be the most comprehensive and holistic framework for thinking about QoL outcomes.

Table 2 summarizes the mapping for the 20 identified frameworks or tools onto the Schalock QoL Domains.

**Table 2:** Mapping of the domains, dimensions, and indicators of each of the framework identified in the research to the schalock et al. (2002) Quality of life domains.

Framework/tool	To which Schalock et al. QoL domains could at least some domains or indicators be mapped?							
	PD	IR	R	SI	SD	MW	PW	EW
*Frameworks where whole domains could be mapped*								
Bigby et al. (2014, Australia)	✓	✓	✓	✓	✓	✓	✓	✓
The Quality Cube (Netherlands)	✓	✓	✓	✓	✓	✓	✓	✓
ASCOT—Social Care related quality of life (UK and internationally)	✓	✓	✓	✓	✓	✓	✓	✓
Changing our Lives Quality of life Standards (UK)	✓	✓	✓	✓	✓	✓	✓	✓
Social Services Quality Standards (Czechia)	✓	✓	✓	✓	✓	✓	✓	✓
Personal Outcomes Measure (the U.S. and internationally)	[✓]	✓	✓	✓	✓			✓
National Quality Forum framework AND the Home and Community-based Services Outcomes (the U.S.)	✓	✓	✓	✓	✓	[✓]		
National Core Indicators (the U.S.)	✓	✓		✓	✓	[✓]		✓
Quality of life Outcomes Domain Framework (Ireland)	✓	✓	✓	✓	✓	[✓]	✓	✓
***Frameworks where individual indicators, standards or parts of domains could be mapped***								
National standards for residential services for children and adults with disabilities (Ireland)	✓	✓	✓	✓	✓		✓	✓
EQUASS (Europe)			✓	✓	✓		✓	
Guidance on a Human Rights-based Approach in Health and Social Care Health Services. By Health Information and Quality Authority (Ireland)			✓	✓	✓		✓	
National longitudinal Transition Study (Shrogren et al) the U.S.	✓	✓			✓	✓	✓	✓
Standards New Zealand Health and disability services standard NZS 8134: 2021	✓	✓	✓	✓	✓	✓	✓	✓
Quality of life impact of services tool (QOLIS) (Europe)	✓	✓		✓	✓	[✓]		✓
Šiška et al. (2021, Czech Republic)	✓	✓	✓	✓	✓	✓	✓	✓
National Standards for Disability services (Australia)	✓		✓	✓	✓		✓	
NDIS Practice Standards and Quality Indicators (Australia)		✓	✓		✓	✓	✓	✓
Person-centred advocacy, vision, and education (the U.S.)	✓	✓	[✓]	✓	✓	[✓]	✓	[✓]

SD, self-determination; MW, material well-being; R, rights; PD, personal development (including meaningful occupation); PW, physical well-being; SI, social inclusion; IR, interpersonal relationships; EW, emotional well-being; [✓], link is indirect or related to one indicator only (e.g., employment).

These eight quality of life domains were therefore adopted to structure the outcomes element of Service Quality Framework (22). The potential indicators in each domain were derived from a number of sources: (1) the frameworks identified in the mapping review and template synthesis above; (2) the wider published literature and theory related to quality of life; and (3) what people with disabilities have said is important to them for a good life. Outcome indicators were provided as both subjective indicators (what people would say when asked) and objective indicators (“what you would see or hear”). Table 3 provides an example of what this looked like for one of the quality of life domains—self-determination. In total there were 47 subjective indicators and 68 objective outcome indicators proposed.

**Table 3:** Example of how the framework was set out, showing one quality of life domain (self-determination) and the corresponding indicators.

QoL domain	Self-reported indicators—what we would like the people in receipt of services to say?	Objective indicators—what would we see and hear?
*Self-determination/autonomy*	• I have choice and control over the big things in life—where I live, who I live with, where I work, how I spend my money, who provides my support and what they help me with.	• Individuals are offered the opportunity and supported to express preferences and make choices about day-to-day aspects of their lives. • Staff use appropriate communication to support choice and respect people's decisions. • People's choices and preferences guide what staff do rather than staff preferences and agendas. • People are helped to understand and predict what their day will be like. • Individuals are supported to understand what is involved in bigger life decisions, with information provided in an accessible way. • Where people might find it difficult to make such decisions, services ensure that the person's will and preference, based on experience of supporting the individual over time as well as their previous choices and decisions, is used to guide decisions. • Individuals have access to independent help such as an advocate to ensure their views are heard.
	• I have control over my day-to day life—what I do, where I go, what I eat and drink, when I do things, how I do things.	
	• I am provided information about choices, decisions, or opportunities in a form that I understand.	
	• I have a way to communicate my needs, wishes and decisions that works for me.	
	• People listen when I tell them or show them what I want.	
	• I have help (e.g., an advocate), if I need it, to let others know what I want and need.	
	• I attend meetings about my care and support and am involved in planning my life and my support.	• Individuals are involved in a meaningful way in identifying goals and aspirations during planning processes. • Individuals are supported to be attend and participate in their planning meetings.
	• I get help to achieve the goals I want to achieve.	
	• I am treated as an individual.	• People are treated as individuals rather than being seen as part of a group of “residents” or “service users”. They are not “forced” to do things with others because of how the service is organised.

#### 2.1.6. Testing the content and face validity of the service quality framework

The Framework and full set of indicators were consulted on with a wide range of knowledge experts who provided feedback from a range of different perspectives. Stakeholders included members of the EASPD task force group on disability service quality, other service providers, academics, representatives of disabled peoples' organizations and family members of people with IDD. Stakeholders came from a range of different countries across Europe as well as more widely. Several elements of the Delphi technique were used during the process to arrive at a group opinion. These included an online survey, individual consultation *via* email or in person and discussion or individual feedback *via* the group facilitator following a presentation. The feedback provided by the stakeholders was systematically analyzed and considered during preparation of the final set of proposed indicators. More information on the findings from the consultation and the detailed resulting framework with all indicators can be found in Šiška and Beadle-Brown (22). Identifying the indicators of transition success.

In terms of indicators of transition success, this paper primarily draws on a mapping of literature, policy and practice in four countries (USA, UK, Czech Republic and Australia). This mapping study combined two methods—template syntheses and rapid literature review. The five members of the research team working on this were all researchers with national and international expertise in the field. Each member drew on their existing knowledge of policy, practice and research on transition in their respective countries and conducted a rapid literature search to identify further resources relevant to transition in their country. Search terms to identify both peer reviewed and grey literature were kept broad (for example, disab* and transition). A template to collate and summarize the information gathered was developed collaboratively and completed by each member of the team for the relevant country.

These four templates were then reviewed by one member of the team who extracted key information into an Excel spreadsheet so that it could be synthesized across countries. The elements most relevant to this paper related to: how transition is defined or conceptualized; models that support successful transition; and the focus of research on transition within each country. As part of the extraction process, gaps in information were identified and the experts asked to add missing information specific to the identified gaps.

## 3. Linking the indicators of transition success to the service quality framework

In this section we will expand and reflect on some of the specific outcomes-based domains and indicators from the Service Quality Framework described above and identify their potential relevance to conceptualizing and supporting successful transition. We will consider how it might be helpful to think about successful transition as the young person moving towards having similar opportunities and QoL as adults without disabilities living in the same community/society. This doesn't mean that everyone's life is the same, but that people have the same opportunities to explore, and then to follow, what is important to them and what they need to do to achieve the things that are important to and for them.

Table 4 provides a summary of the mapping of the transition success indicators identified in the literature and the QoL domain indicators from the Service Quality Framework.

**Table 4:** Summary of transition success indicators and the QoL domain indicators.

Indicators of transition success	QoL Domains
Having a job (employment) Financial independence	Direct: Personal development (including meaningful occupation). Material well-being, security Indirect: Emotional well-being Social relationships Social inclusion
Independent living/moving out of the family home	Material well-being Rights Self-determination/autonomy
Further education	Personal Development
Growing your social networks, relationships and being part of your community	Social relationships Social inclusion
Physical and mental health/well-being	Physical well-being Emotional well-being *These two effect on people's ability to do some of the things that impact on other elements of QoL.*

### 3.1. Transition success indicator: employment and financial independence

Considering first the transition indicator of having a job (employment). Employment is considered important in several ways—it is widely recognized that having meaningful ways to spend your time is good for both *personal development* and *emotional well-being*. Also important for emotional well-being is the structure and routine that having a job often gives. Of course, paid employment is also important for *material well-being,* the ultimate level of which would be financial independence. Finally, paid employment is also considered important in many societies as a way of contributing to society—e.g., by paying taxes, national insurance etc.- thus employment can also be important for people to be seen as active citizens, accessing their *rights* and being *socially included*. This has the unfortunate effect of setting up those who are not able to take up paid employment for health or disability reasons or because of caring responsibilities, in a negative light. There are many barriers to young people with IDD accessing and keeping employed positions, many of which are nothing to do with the needs, skills, and motivation of the individuals themselves (18). Although around two thirds of people with learning disabilities in the UK report that they would like to be in paid employment, Mencap's 2019 survey in the UK found that only 23% percent of people with intellectual disability (aged 18–64) have a paid job and for 62% of those with a paid job, they worked for 16 h a week or less.^1^ Although 77% of autistic adults in the UK want paid employment, the Office of National Statistics report on Outcomes for Disabled People in the UK 2020, found that autistic people are the least likely to be in work than any other disabled group with just under 22% in employment. Even in the US where the focus is more on transitioning to employment, only 34% of people with ID (aged 21–64) are employed and approximately half of these work in a sheltered setting rather than in open employment (29).

Lecerf (30) notes that just over half of people with a disability are employed compared to three quarters of people without disabilities. Women with disabilities, young disabled people and those with high support needs are the most likely to be excluded from the labour market. Vaalavuo (31) commented that an increasing number of Europeans are working part-time. However, for persons with disabilities part-time work might be the only available option due to health issues or/and work-limitations. In addition to decreasing availability, part-time jobs are often of lower quality with lower hourly wages, provide poorer training and career opportunities, and, in the long run, reduce pension entitlements.

Even once they have got a job, retaining that job is often an issue (32). Education and training programs related to employment do not always result in jobs for people (18). In many countries, there is also what is sometimes called the “benefit trap”—where earning a salary can mean people lose their benefits and regaining benefits is extremely difficult to do should someone lose their job or find they cannot cope with the job they took on. These issues have been accentuated by the financial crisis and the COVID pandemic (29, 33). Lack of accessibility of environments, transport, communications in the workplace, lack of structure and guidance can also have negative impact—ensuring people have reasonable accommodation is a key part of the *Rights* domain of QoL.

Another issue that can limit the possibilities for people to access paid work, is the limited range of jobs that are sometimes considered as suitable or accessible for individuals with IDD. Examples of creative approaches we have come across in practice include options such as developing a small business (e.g., a window cleaning, car cleaning business, catering business, gardening services, dog walking business); job sharing (for example a newspaper round) amongst those who live together; being a local rep for a catalogue company; providing office services such as shredding, copying etc.

Whilst supporting young people to access paid employment in a way that ensures their needs are met is clearly desirable, focusing on other ways to ensure personal development, social inclusion and emotional wellbeing whilst looking for paid employment is also really important—voluntary work, helping out neighbors or looking after pets, caring for their own home and garden, growing fruit and veg to help save money on shopping, making things like cards, presents, baking for friends, family or charities, taking part in sports and other leisure activities, being part of clubs and groups such as choirs, art groups, dance troupes, theatres, etc.

### 3.2. Transition success indicator: independent living/moving out of the family home

Living independently doesn't necessarily mean that you are living on your own and without support. It is about, at the same age as most of your peers, moving into a home you consider your own, even if you are sharing with others, with the support you need to have your needs met and to participate in your local community as fully as possible. It is about having choice over where you live and with whom you live and not having your support tied to your place of living so that you can move and take your support with you, or you can change who provides your support without having to change where you live. Of course, the age at which this happens for young people without IDD varies by country, culture, financial status etc. However, those with IDD are more likely to remain living in their family home or to move into congregate settings than even those with other disabilities (34). In terms of choice, there is little research on choice over living situation and support arrangements and most of what there is more than 10 years old. However, the literature that does exist suggests that the majority of people with intellectual disabilities do not experience choice and control over living arrangements or support arrangements (3, 35–37).

In terms of mapping to the QoL outcome domains and indicators, moving out of the family home and living independently in the community, with choice about where and with whom you live and who provides your support, is an indicator of the QoL domains of *personal development*, *material well-being* and *self-determination*.

### 3.3. Transition success indicator: further education

This element of transition success is most clearly linked to the QoL domain of “*personal development*”. It is most commonly associated with formal processes such as attending adult education classes, college or university, but also participating in an internship or apprenticeship. Being able to attend the same further education venues as your peers is an important right but also is associated with barriers in terms of knowledge and attitudes of teachers, accessibility of environments, etc. In addition, personal development can also be achieved through many more informal opportunities to learn and to practice skills you already have so that you develop and experience success. This in turn is related to “*emotional well-being”*, in particular self-esteem and confidence.

### 3.4. Transition success indicator: growing your social networks, relationships and being part of your community

Needing no detailed explanation, these indicators of transition success are clearly linked to the QoL domains of “*social relationships*” and “*social inclusion*”. Thinking about these domains as broadly as possible can facilitate people to come into contact with a wider range of people in the community more often, can help change attitudes towards people with IDD when people are seen contributing to society in some way and allow people to show their skills and personalities. This in turn may open doors to opportunities for employment, new relationships, and new ways to be part of society and increase people's sense of belonging and emotional well-being.

However, at an even more basic level, young people need to feel they can trust those who provide support for them especially at this relatively traumatic time. So just ensuring young people are being listened to, respected, and have the freedom and support to make decisions about relationships is a key aspect of becoming an adult.

### 3.5. Transition success indicator: physical and mental health/well-being

We have identified above several ways that other elements of transition may be connected to emotional well-being. However, it is also important to ensure that people's physical and mental health is being promoted and protected as much as possible in order to ensure they are able to engage with opportunities for occupation, participation, relationships, inclusion etc. If people's health care needs are not being met, then holding down a job is likely to be relatively impossible for them. A key point here, however, is the fact that the process of transition is seen as a very stressful one for both young people and their families (38, 39). This is particularly true for young people who are autistic (40). Putting things in place to make the process as easy as possible for both will ensure people start off on a “good foot” in terms of adult life.

### 3.6. Additional elements of transition to adulthood—decision making and autonomy

One important element of becoming an adult that is rarely explored in research to date is the issue of supporting independence in decision making, legal capacity, having personal relationships, having a family and how we can prepare young people with IDD for those events and opportunities. For many young people with IDD they may not have very much decision making experience by the time they legally become an adult and they may have little experience of different options for work, living, education, activities, etc. to help them make decisions. When thinking about whether people are becoming self-determined adults, then the QoL Framework gives us some indications of how we would know whether this was happening. It also sensitizes those who provide support to know what they should be aiming to help people achieve and experience (See Table 2).

## 4. Conclusion

This paper set out to discuss the potential application of an outcomes-based Framework focused on the Quality of Services for people with disabilities to the conceptualization and evaluation of successful transition. We have proposed that using a the framework by mapping its indicators onto the QOL domains could potentially provide a more holistic, comprehensive and inclusive way of examining transition success and at the quality of transition services. Whilst indicators such as employment and further education are important, so are people's experiences while accessing these and so are good outcomes in other domains. For example, someone could have a paid job but continue to live in a larger institutional setting with no choice about where they live and who they live with or on what to spend their money. Or someone could go to college and do a course they are interested in but find it very stressful and experience bullying while there. For some people, finding paid jobs in the open market or a place at mainstream college will be much harder and take longer to arrange, more funding to support etc. However, this doesn't mean that they can't experience a wide range of opportunities for meaningful occupation that improve all other QoL domains and may even lead to an income with enough creativity from those who provide support.

If this QOL focused Service Quality framework was to be adopted as a way to judge whether young people have successfully transitioned to adulthood (taking account of cultural differences and individual preferences) or to judge the quality of transition services, then this would have a number of implications.

### 4.1. Implications for research

Firstly, although the original Schalock et al. (24), Quality of Life framework used to organize and structure the outcomes elements Service Quality Framework used for this theoretical discussion, is a well-established and validated framework, the Šiška and Beadle-Brown (22) Service Quality Framework still needs to be empirically tested. The original development work on the Service Quality Framework explored face and content validity, but establishing the feasibility, reliability and other aspects of validity of the framework to allow service providers, quality assessors and researchers to use it to measure service quality is still needed. Although there are a number of existing subjective measures looking at the QOL outcome domains, there are few tools that allow assessment of the objective indicators. Future research should prioritize establishing the feasibility, useability and reliability of the Service Outcomes framework. Such research could usefully include services supporting young people with IDD leading up to and through the transition from school, allowing the validity of the suggestions made in this discussion paper to be tested empirically.

Secondly, as it would be a more holistic and wider view of transition success, such a framework could potentially allow more people with IDD to be evaluated to be experiencing successful transition to adulthood in more life areas, even if they are not working or attending post-secondary education. However, this would require the use of a wider range of research measures, both subjective and objective, with evidence of validity and reliability. Some elements of the framework are likely to be best evaluated using observational measures, which carries implications in terms of project duration, costs, and potentially ethical approval. However, observational methods are already well established in the field of IDD research and quality evaluation (41) with particular importance when gathering the experiences of people with more severe intellectual disability.

### 4.2. Implications for practice

The use of such a wide and holistic framework for conceptualizing transition is likely to mean that a greater number of agencies would need to be involved, working in partnership, and over a longer period of time, with a greater focus on starting transition planning and preparation for adulthood at an earlier stage.

Supporting transition to adulthood is an ongoing process and needs to be built up over quite a long time. This would mean that schools and potentially families and children's services would have an even more important role in preparing young people for adult life and would potentially require curriculum and support content to be modified. Families are likely to need support as they rarely have access to the training and other forms of support available to staff in schools and other services. They may also have been led by professionals and others to have low expectations of their son or daughter and may need help to see the potential the person has.

Although there is not a lot of literature focused on the factors that bring about successful transition outcomes, the research that does exist suggests that key factors might include young people having experience of different jobs to help them decide what they might like to do after school (42) and good co-ordination between educational system and the labour marker (18). In transition from child to adult health services, Kerr et al. (43) found validating evidence for three of the eight interventions reviewed—an early start to the transition process, developing adolescent/young adult autonomy and the role of parents/carers. The importance of effective communication between healthcare professionals and the adolescent/young adult and their parents/carers was also highlighted. It is conceivable that these interventions are much more general and not specific to health contexts and this tie into the findings from Garrels and Sigstad (15).

The frequent focus of the literature has been on good transition planning. However, planning on its own is not enough (15). Some literature has suggested that giving people a transition related personal budget can be useful. However, having access to funding is only any use if you know what you want to buy and you have a range of good quality options from which to purchase. Looking at the wider literature in terms of improving people's QoL is helpful here—to improve people's quality of life, we know that the nature of the support provided is key (see, for example 41). Support needs to be enabling and empowering, giving people many opportunities to engage in meaningful activities and interactions in ways that are manageable for them, providing just enough of the right support so that people can gain the experience they need to make choices and decisions, can develop their skills, and can become a full and active citizen. For many people this needs to happen consistently over quite a long period of time.

To ensure young people experience successful outcomes as they transition to adulthood, schools, colleges, and transition support services where they exist would need to be paying attention to all of these things. This may require changes in the training of teachers and staff at transition services. It also may require changes in policy and resource planning and allocation systems. Systems and frameworks used to assess quality may also need to be adapted. However, having a QoL based framework for measuring quality of services supporting young people and for adult support settings, may help to reduce the gap or indeed the steep divide that often exists when young people reach 18 (17). It might also help to reduce the experience of families coming up against a “cliff edge” or of entering a “black hole” (44).

## Footnotes

^1^ https://www.mencap.org.uk/about-us/what-we-think/employment-what-we-think

## References

[1] Ravenscroft J Wazny K Davis JM. Factors associated with successful transition among children with disabilities in eight European countries. PLoS One. (2017) 12(6):e0179904. 10.1371/journal.pone.0179904 28636649 PMC5479584

[2] United Nations Convention on the Rights of Persons with Disabilities (December 13, 2006).

[3] Šiška J Beadle-Brown J Káňová Š Šumníková P. Social inclusion through community living: current situation, advances and gaps in policy, practice and research. Social Inclusion. (2018) 6(1):94–109. 10.17645/si.v6i1.1211

[4] Ormston R Eunson J McAteer G. Improving outcomes for people with learning disabilities: Opportunities and challenges for housing. Key Findings and Recommendations. Ipsos MORI Scotland (2017). Available at: https://www.scld.org.uk/wp-content/uploads/2017/10/Improving-Outcomes-key-findings.pdf

[5] Kaehne A Kiernan J Ridley J. Systematic review of study designs and methods in health transition research for young people with intellectual disabilities. Heliyon. (2019) 5(11):e02750. 10.1016/j.heliyon.2019.e02750 31768431 PMC6872843

[6] Sullivan WF Heng J Abells D Perry A Henze M. Supporting adults with intellectual and developmental disabilities to cope and thrive through transitions to later-life phases. Can Fam Physician. (2019) 65(Suppl 1):S30–2. 31023777 PMC6501712

[7] Mazzotti VL Rowe DA Kwiatek S Voggt A Chang W Fowler C Secondary transition predictors of postschool success: an update to the research base. Career Dev Transit Except Individ. (2021) 44(1):47–64. 10.1177/2165143420959793

[8] Wehman P. Life beyond the classroom: Transition strategies for young people with disabilities. 3rd edn. Baltimore: Paul H. Brookers Publishing (2001).

[9] Wehman P Schall C Carr S Targett P West M Cifu G. Transition from school to adulthood for youth with autism Spectrum disorder. J Disabil Policy Stud. (2014) 25(1):30–40. 10.1177/1044207313518071

[10] Settersten RA Ray B. What's going on with young people today? The long and twisting path to adulthood. Future Child. (2011) 20:19–41. 10.1353/foc.0.0044 20364620

[11] Dispenza F. Empowering the career development of persons with disabilities (PWD). J Career Dev. (2019) 48(5):670–85. 10.1177/0894845319884636

[12] Morwane RE Dada S Bornman J. Barriers to and facilitators of employment of persons with disabilities in low- and middle-income countries: a scoping review. Afr J Disabil. (2021) 10:833. 10.4102/ajod.v10i0.833 PMC8252133 34230882

[13] Šiška J Tichá R Stancliffe R Beadle-Brown J Abery B Káňová Š. Transition to where and to what: mapping the current situation, advances and gaps in policy, practice, and research on transition for students with IDD across four countries. Submittted to the Journal of Policy and Practice in Intellectual Disability.

[14] France A Threadgold S. Youth and political economy: towards a bourdieusian approach. J Youth Stud. (2016) 19(5):612–28. 10.1080/13676261.2015.1098779

[15] Pearson C Watson N Gangneux J Norberg I. Transition to where and to what? Exploring the experiences of transitions to adulthood for young disabled people. J Youth Stud. (2021) 24(10):1291–307. 10.1080/13676261.2020.1820972

[16] Bradshaw J. Ensuring quality in practice—person-centred approaches. In: Šiška J Beadle-Brown J, editors. The development, conceptualisation and implementation of quality in disability support services. Prague: Karolinum Press (2021).

[17] Maniatopoulos G Le Couteur A Vale L Colver A. Falling through the gaps: exploring the role of integrated commissioning in improving transition from children's To adults’ services for young people with long-term health conditions in England. J Health Serv Res Policy. (2018) 23(2):107–15. 10.1177/1355819617752744 29475369 PMC5901047

[18] Garrels V Sigstad HMH. Employment for persons with intellectual disability in the nordic countries: a scoping review. J Appl Res Intellect Disabil. (2021) 34:993–1007. 10.1111/jar.12880 33709541

[19] McNeil B Reeder N Rich J. A framework of outcomes for young people. UK: The Young Foundation (2012). https://youngfoundation.org/wp-content/uploads/2012/10/Framework-of-outcomes-for-young-people-July-2012.pdf

[20] Test DW Mazzotti VL Mustian AL Fowler C Kortering L Kohler P. Evidence-Based secondary transition predictors for improving postschool outcomes for students with disabilities. Career Dev Except Indi. (2009) 32(3):160–81. 10.1177/0885728809346960

[21] Lindsay S Lamptey D-L Cagliostro E Srikanthan D Mortaji N Karon L. A systematic review of post-secondary transition interventions for youth with disabilities. Disabil Rehabil. (2018) 41(21):2492–505. 10.1080/09638288.2018.1470260 29726294

[22] Šiška J Beadle-Brown J. (Tech.). Innovative frameworks for measuring the quality of services for persons with disabilities. Brussels, Belgium: EASPD (2021).

[23] Donabedian A. Explorations in quality assessment and monitoring. Health Administration Press (1980).

[24] Schalock RL Brown I Brown R Cummins RA Felce D Matikka L Conceptualization, measurement, and application of QoLfor persons with intellectual disabilities: report of an international panel of experts. Ment Retard. (2002) 40(6):457–70. 10.1352/0047-6765(2002)040<0457:cmaaoq>2.0.co;2 12408748

[25] Schalock RL Verdugo MA. Quality of life as a change agent. In: Brown RI Faragher RM, editors. Quality of life and intellectual disability: knowledge application to other social and educational challenges. New York: Nova Science Publishers (2014). p. 19–34.

[26] Šiška J Beadle-Brown J. Innovative framework for measuring the quality of services for persons with disabilities. EASPD. International conference: quality of life & support services: from words to action; October 13, 2022; Malta (n.d.).

[27] Peters MDJ Godfrey C McInerney P Munn Z Tricco AC Khalil H. Chapter 11: scoping reviews (2020 version). In: Aromataris E Munn Z, editors. JBI Manual for evidence synthesis. Prague: JBI (2020). Available at: https://synthesismanual.jbi.global. 10.46658/JBIMES-20-12

[28] Šiška J Beadle-Brown J. The development, conceptualisation and implementation of quality in disability support services. Karolinum Press (2021).

[29] Siperstein GN Parker RC Drascher M. National snapshot of adults with intellectual disabilities in the labor force. J Vocat Rehabil. (2013) 39(3):157–65. 10.3233/jvr-130658

[30] Lecerf M. Employment and disability in the European Union—European Parliament (n.d.). Available at: https://www.europarl.europa.eu/RegData/etudes/BRIE/2020/651932/EPRS_BRI(2020)651932_EN.pdf (Retrieved April 10, 2022)

[31] Vaalavuo M. Part-time work: A divided Europe. Employment, Social Affairs & Inclusion—European Commission (2016). Available at: https://ec.europa.eu/social/main.jsp (Retrieved March 10, 2022)

[32] Kocman A Fischer L Weber G. The Employers’ perspective on barriers and facilitators to employment of people with intellectual disability: a differential mixed-method approach. J Appl Res Intellect Disabil. (2018) 31:120–31. 10.1111/jar.12375 28585231

[33] Linehan C Birkbeck G Araten-Bergman T Baumbusch J Beadle-Brown J Bigby C. COVID-19 IDD: findings from a global survey exploring family members’ and paid staff's Perceptions of the impact of COVID-19 on individuals with intellectual and developmental disabilities (IDD) and their caregivers. HRB Open Res. (2022) 5:27. 10.12688/hrbopenres.13497.1 35615436 PMC9111363

[34] Šiška J Beadle-Brown J. Progress on deinstitutionalisation and the development of community living for persons with disabilities in Europe: are we nearly there? Disabil Soc. (2022):1–20. 10.1080/09687599.2022.2071676

[35] Stancliffe RJ Lakin KC Larson S Engler J Taub S Fortune J. Choice of living arrangements. J Intellect Disabil Res. (2011) 55:746–62. 10.1111/j.1365-2788.2010.01336.x 21029234

[36] Hatton C Watters J. National personal budgets survey 2013: summary of main findings and next steps. Lancaster: Lancaster University and In Control (2013).

[37] Söderström S Tøssebro J. Innfridde mål elelr brudte visjoner [fulfilled ideals or broken promises]. Trondheim: NTNU Social Research (2011).

[38] Codd J Hewitt O. Having a son or daughter with an intellectual disability transition to adulthood: a parental perspective. Br J Learn Disabil. (2020) 49(1):39–51. 10.1111/bld.12327

[39] Biswas S Tickle A Golijani-Moghaddam N Almack K. The transition into adulthood for children with a severe intellectual disability: parents’ views. Int J Dev Disabil. (2016) 63(2):99–109. 10.1080/20473869.2016.1138598

[40] Hendricks DR Wehman P. Transition from school to adulthood for youth with autism Spectrum disorders: review and recommendations. Focus on Autism Other Dev Disabil. (2009) 24(2):77–88. 10.1177/1088357608329827

[41] Mansell J Beadle-Brown J. Active support enabling and empowering people with intellectual disabilities. Jessica Kingsley Publ (2012).

[42] Bigby C De Losa L. After-school jobs for students with intellectual disabilities. La Trobe. Report (2021). doi: 10.26181/60876b527487b

[43] Kerr H Widger K Cullen-Dean G Price J O'Halloran P. Transition from children's To adult services for adolescents/young adults with life-limiting conditions: developing realist programme theory through an international comparison. BMC Palliat Care. (2020) 19(115):1–11. 10.1186/s12904-020-00620-2 32731863 PMC7393825

[44] Broach S Clements LJ. Disabled children: a legal handbook. Legal Action Group (2020).

